# Annual Address of the Retiring President of the Buffalo Medical Association

**Published:** 1858-08

**Authors:** Austin Flint


ART. II.—*Annual Address of the Retiring President of the Buffalo Medical Association.* Delivered in June, 1858, by AUSTIN FLINT, M. D.

GENTLEMEN OF THE ASSOCIATION:

In compliance with a by-law of our association which makes it incumbent on the retiring president to deliver an address on some medical or surgical subject, I invite your attention to a few desultory thoughts on *Conservative Medicine.* I am indebted for this term to my friend and colleague, Prof. Hamilton. On a convivial occasion some years since, I had the honor to propose as a toast, *conservative surgery*, exemplified by the principles and practice of the distinguished gentleman just named, and in responding to this toast he claimed that conservatism is a principle not less important to the physician than the surgeon; that conservative medicine is as much a reality as conservative surgery, and that the former, as well as the latter, is to govern, within certain limits, the conduct of the practitioner. The term may guide us to some reflections which will not be without interest and value.

What meaning is to be attached to the expression "conservative medicine?" We understand by "conservative surgery" a reluctance to mutilate the body by surgical operations; a judicious expectancy which looks constantly toward cure without resort to the knife, not repudiating the latter, but resorting to it only as an alternative rendered necessary by insufficiency of the means of restoring or maintaining the integrity of the organism. The conservative surgeon directs his efforts first and chiefly to the preservation of the body with all its parts, and when these efforts become ineffectual and longer delay dangerous, he does not hesitate to sacrifice portions of the corporeal fabric, more than the prudent mariner hesitates to throw overboard a rich cargo to prevent his ship from sinking, or the fireman to pull down valuable buildings to stay the progress of a conflagration. Conservative medicine, in like manner, is reluctant to impair the conditions on which depend life and health. It accepts the maxim of Chomel, that the first object in the mind of the medical practitioner should be not to do harm; the second object being to endeavor to do good. The conservative physician seeks, above all things, not to compromise, except when absolutely necessary, and only to a necessary extent, the vital forces; in directing his attention to disease, he never shuts out of view the system at large; his aim is not merely to destroy maladies, but to effect recovery with the powers of the constitution as complete as possible. A brief definition of conservative medicine is not practicable. This comparison will serve to give a general idea of the significance of the expression.

The knife of the surgeon is manifestly a calamitous necessity; and it is accepted with gratitude because it is a substitute for other and greater calamities. The same is true of medication. A medicine is always an evil; it becomes a remedy in relieving of other and greater evils. The fact as regards surgery is clear enough; in its application to medicine it is less clearly seen, and, hence, is not only overlooked by the vulgar, but, to a vast extent, by medical practitioners. A remedy, not less than the scalpel, is an alternative. It is not to be inferred from the existence of disease, that remedies are to be employed, more than that every swelling is to be cut out, as a matter of course. The use of remedies should always imply that recovery from disease will not take place so certainly, so speedily or so perfectly without, as with them. If this be not assumed, it would be as absurd to subject the system to medication, as it would to make an incision into the flesh without any definite object. I speak now, of course, not of simple palliative remedies, but of those which exert some decided effect on the economy beyond the mere alleviation of present symptoms. In another point of view, a potent medicine may be compared with a surgical operation; not only is it either indicated or not indicated, and therefore useful or not useful, but it must either do good or harm. A remedy, in proportion to its activity, is potent for evil if not for good. A useless amputation is something more than an act of supererogation. This statement needs no argumentation. A salivation or a venesection when not needed, is not less entitled to be considered as involving an irrecoverable loss of a certain amount of vital force, although the fact is not always so distinctly appreciable. The system has lost somewhat of its power of endurance and resistance after being subjected to any unnatural disturbing agency. An active remedy when not required is always a perturbatory agent. If it be indicated, it may save life and secure the recovery of health; but if not needed, as an alternative by which a greater evil is avoided, it inflicts an injury on life and health not the less real and irreparable because slight and inappreciable.

Conservative medicine adopts as a principle of action, renunciation of all medication not sanctioned clearly by reason or experience, on the ground of its being useless and pernicious. This rule is only deviated from when, with proper care and judgment, and under appropriate restrictions, remedial measures are employed experimentally in order to advance our knowledge of therapeutics. Self evident as is the correctness of this principle when viewed as an abstract proposition, a glance at the history of medicine suffices to show that it has not had its legitimate effect on medical practice. The natural philanthropic impulses of our nature interfere with it. It is difficult to forbear endeavors to remove disease even at the risk not only of failure, but of doing harm to the patient if we do not succeed. The patient is often anxious to incur this double risk, and desires to submit to measures the potency of which is proportionate to the gravity of the malady. Another obstacle is the want of definite knowledge respecting the natural tendencies of diseases. This knowledge is the true point of departure for the study of therapeutics. Before we are prepared to judge of the influence of remedies in arresting or abridging any disease, we should know what will be its probable issue and duration without active medication. Strange as it is, the importance of the natural history of diseases in this aspect has not been fully appreciated, and at this moment we are acquainted with the intrinsic tendencies of few diseases sufficiently to be able to judge correctly concerning the extent to which they are controllable by art. The knowledge, however, which has within late years been acquired of the history of certain affections when left to run their course without active medication, has influenced greatly therapeutical notions. Nearly twenty-five years ago, Prof. Jacob Bigelow, of Boston, published a brief paper on the self-limited character of certain diseases. The enumeration of the simple truth that some affections have a defined career, ending after a certain duration in accordance with a limitation inherent in themselves, marked the beginning of a change in views and practice in New England almost revolutionary, and this change extended thence, more or less, over our country, so that when some years afterward Dr. Forbes, of London, published his papers entitled Young Physic, they contained little that was new or striking to the intelligent American medical reader.

Conservatism as a predominant principle in the management of disease, is, in fact, a characteristic of the present epoch in medical history. The preservation of the vital forces is an object in the mind of the judicious practitioner in all cases prominent and often paramount. In the treatment of the continued and eruptive fevers, nearly all the important measures are directed to this end. We do not strive to arrest or to abridge these forms of fever, but to sustain the powers of life till the morbid processes which constitute the disease end by their own limitations. It is, however, but a few years since these fevers were treated by active medication, which, as it was useless, must have been mischievous. The periodical fevers may be controlled by certain remedies, and hence, the first object in these forms of febrile disease, is to resort to the medication which experience has shown to exert a special influence upon them. In the treatment of acute inflammations, conservatism is usually the leading principle. As a type of this class of affections take pneumonia in the adult. A few years ago it would have been culpably hazardous in any practitioner to watch the course of this disease without active interference. Some observers have had the hardihood to do this in a great number of cases, and the result has been that the disease is found to end favorably in a very large ratio of instances when no potent medication is resorted to. Most judicious practitioners at the present time are agreed that in the management of this disease it is safer to forego active interference than to incur much risk of impairing by active measures those powers of the system upon which dependance must be placed for restoration. This remark will equally apply to acute inflammations in general. In chronic maladies, structural lesions, malignant affections, etc., our expectations as regards prolongation of life, and the preservation of those faculties of mind and body which are not directly compromised, are based far more on measures which are conservative rather than remedial. In such cases little may be hoped for from curative efforts, when much may be anticipated from judicious efforts to preserve the powers of life. As an instance illustrating the vast improvement which has been effected by the simple substitution of conservative measures for those which were erroneously supposed to be curative, pulmonary tuberculosis may be cited. They who have been medical observers for the last twenty years, have witnessed a complete revolution in the management of this disease. General and local depletion, mercurialization, cathartics, emetics, counter-irritation, low diet and confinement in an uniform temperature within doors, severally or collectively, were supposed to exert to some extent a curative influence. Nothing can be clearer than that these falsely considered means of cure were positively injurious. Since they have all been abandoned and reliance is had, not on any special curative agent, but on those conservative influences derived from hygiene rather than medication, recovery from the disease is not infrequent, and its progress in fatal cases is far less rapid.

The subject which I have selected for a few remarks offers in various directions abundant scope for reflection and inquiry. I have thrown out, as I

proposed at the outset, only a few desultory thoughts. In conclusion, lest the tenor of these remarks should lead to misapprehension, let me say that there is a false conservatism in medicine as well as in surgery. Therapeutical measures which in their effects are depressing, enfeebling and spoliative, are, nevertheless, under certain circumstances, to be resorted to not less than mutilating surgical operations in certain cases of disease or injury. To determine correctly when these measures are necessary; to regulate their application, carrying them far enough, but not too far; in other words, to understand the limits of true conservatism, knowing when to employ heroic remedies and when to exercise an equal heroism in resisting the natural tendency of the mind to active interference, this is the part of the safe and efficient physician, as it is the part of the safe and efficient surgeon to know when to spare the knife, and when its use is required by science and humanity.

